# Supplementary material for: Anthropogenic Impacts on Bark and Ambrosia Beetle Assemblages in Tropical Montane Forest in Northern Borneo
Source: Insects. 2025 Jan 26;16(2):121. doi: 10.3390/insects16020121 (PMC11855381; doi:10.3390/insects16020121)
Supplement: Supplementary file 1 [file insects-16-00121-s001.zip › Revised_Supplementary Figures_S1 and S2.pptx]

## Slide 1
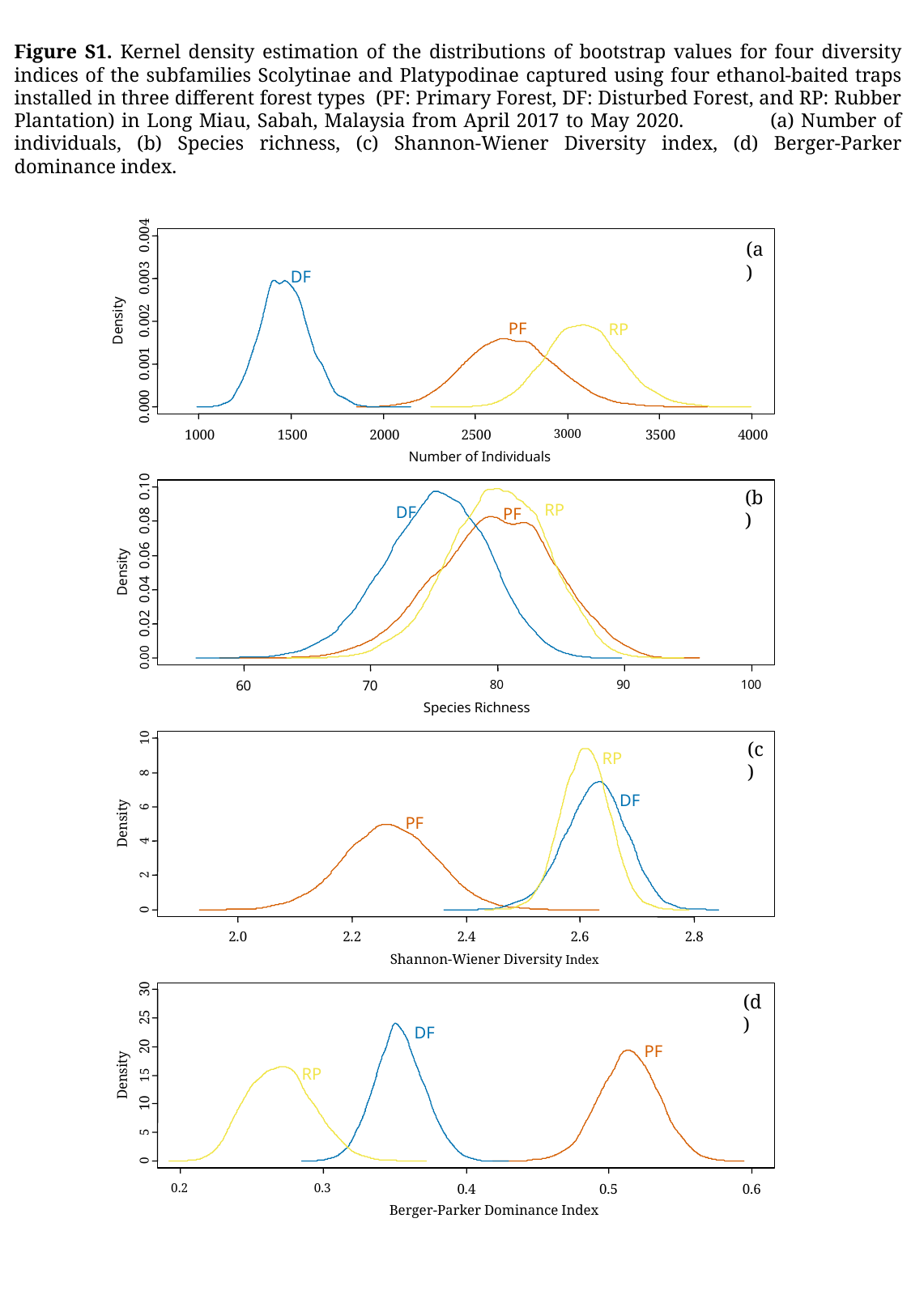

Figure S1. Kernel density estimation of the distributions of bootstrap values for four diversity indices of the subfamilies Scolytinae and Platypodinae captured using four ethanol-baited traps installed in three different forest types (PF: Primary Forest, DF: Disturbed Forest, and RP: Rubber Plantation) in Long Miau, Sabah, Malaysia from April 2017 to May 2020.	 (a) Number of individuals, (b) Species richness, (c) Shannon-Wiener Diversity index, (d) Berger-Parker dominance index.
0.004
(a)
DF
PF
RP
RP
DF
PF
RP
DF
PF
DF
PF
RP
0.003
Density
0.002
0.001
0.000
1000
1500
2000
2500
3000
3500
4000
Number of Individuals
0.10
(b)
0.08
0.06
Density
0.04
0.02
0.00
60
70
80
90
100
Species Richness
10
(c)
8
6
Density
4
2
0
2.0
2.2
2.4
2.6
2.8
Shannon-Wiener Diversity Index
30
(d)
25
20
Density
15
10
5
0
0.2
0.3
0.4
0.5
0.6
Berger-Parker Dominance Index

## Slide 2
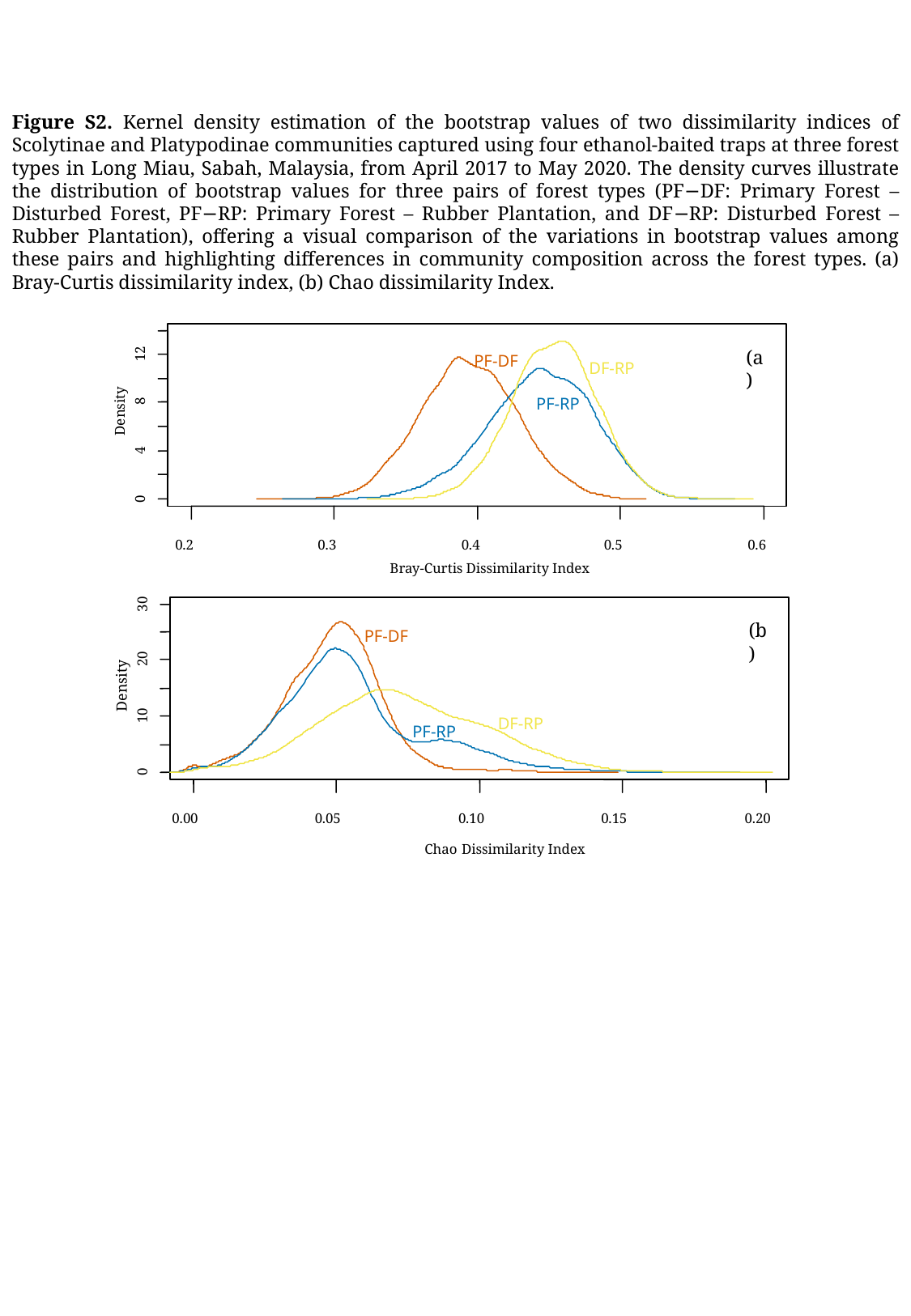

Figure S2. Kernel density estimation of the bootstrap values of two dissimilarity indices of Scolytinae and Platypodinae communities captured using four ethanol-baited traps at three forest types in Long Miau, Sabah, Malaysia, from April 2017 to May 2020. The density curves illustrate the distribution of bootstrap values for three pairs of forest types (PF−DF: Primary Forest – Disturbed Forest, PF−RP: Primary Forest – Rubber Plantation, and DF−RP: Disturbed Forest – Rubber Plantation), offering a visual comparison of the variations in bootstrap values among these pairs and highlighting differences in community composition across the forest types. (a) Bray-Curtis dissimilarity index, (b) Chao dissimilarity Index.
12
8
Density
4
0
0.2
0.3
0.4
0.5
0.6
Bray-Curtis Dissimilarity Index
30
20
Density
10
0
0.00
0.05
0.10
0.15
0.20
Chao Dissimilarity Index
(a)
PF-DF
DF-RP
PF-RP
(b)
PF-DF
DF-RP
PF-RP
