# Supplementary material for: Anthropogenic Impacts on Bark and Ambrosia Beetle Assemblages in Tropical Montane Forest in Northern Borneo
Source: Insects. 2025 Jan 26;16(2):121. doi: 10.3390/insects16020121 (PMC11855381; doi:10.3390/insects16020121)

Figure S1. Kernel density estimation of the distributions of bootstrap values for four diversity indices of the subfamilies Scolytinae and Platypodinae captured using four ethanol-baited traps installed in three different forest types (PF: Primary Forest, DF: Disturbed Forest, and RP: Rubber Plantation) in Long Miau, Sabah, Malaysia from April 2017 to May 2020. (a) Number of individuals, (b) Species richness, (c) Shannon-Wiener Diversity index, (d) Berger-Parker dominance index.

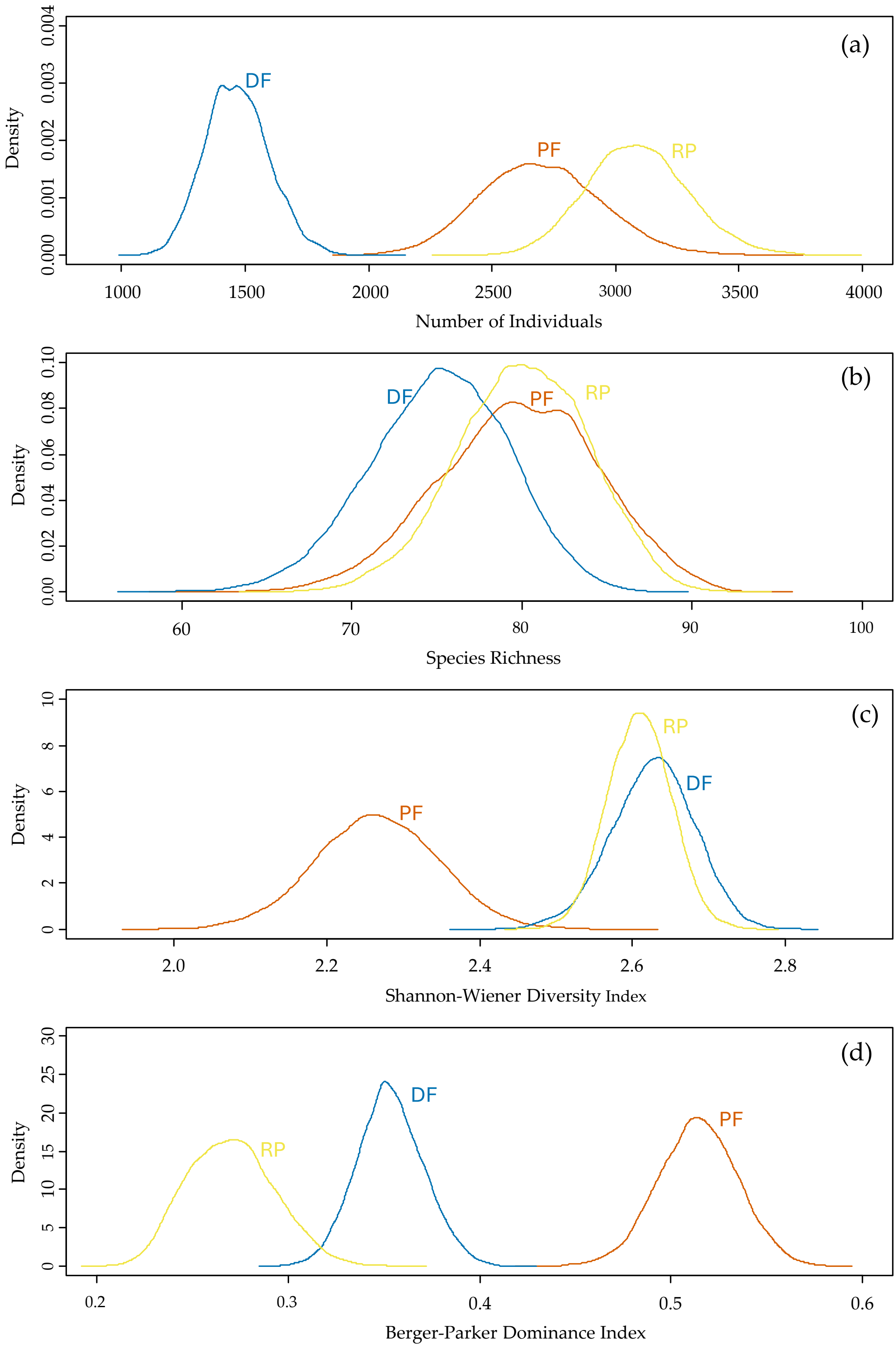

Supplement: Supplementary file 1 [file insects-16-00121-s001.zip › Revised_Supplementary Figure_S1.pdf]
