# Supplementary material for: Anthropogenic Impacts on Bark and Ambrosia Beetle Assemblages in Tropical Montane Forest in Northern Borneo
Source: Insects. 2025 Jan 26;16(2):121. doi: 10.3390/insects16020121 (PMC11855381; doi:10.3390/insects16020121)

Figure S2. Kernel density estimation of the bootstrap values of two dissimilarity indices of Scolytinae and Platypodinae communities captured using four ethanol-baited traps at three forest types in Long Miau, Sabah, Malaysia, from April 2017 to May 2020. The density curves illustrate the distribution of bootstrap values for three pairs of forest types (PF–DF: Primary Forest – Disturbed Forest, PF–RP: Primary Forest – Rubber Plantation, and DF–RP: Disturbed Forest – Rubber Plantation), offering a visual comparison of the variations in bootstrap values among these pairs and highlighting differences in community composition across the forest types. (a) Bray-Curtis dissimilarity index, (b) Chao dissimilarity Index.

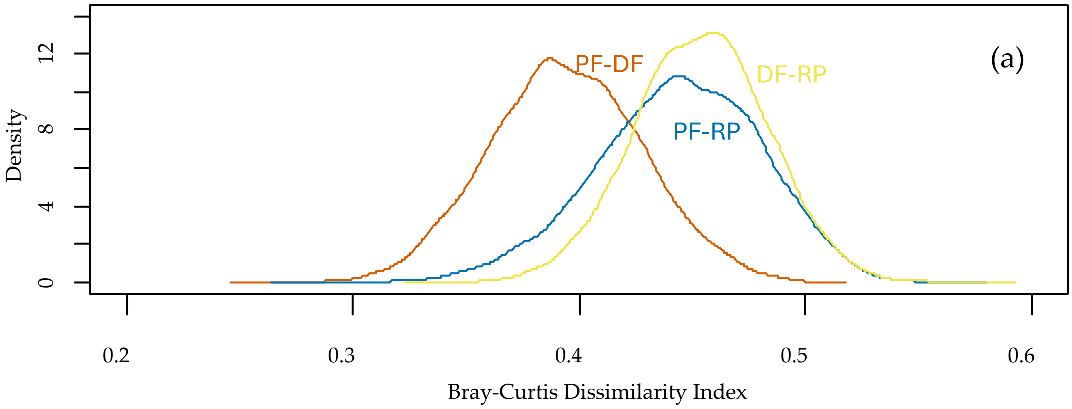

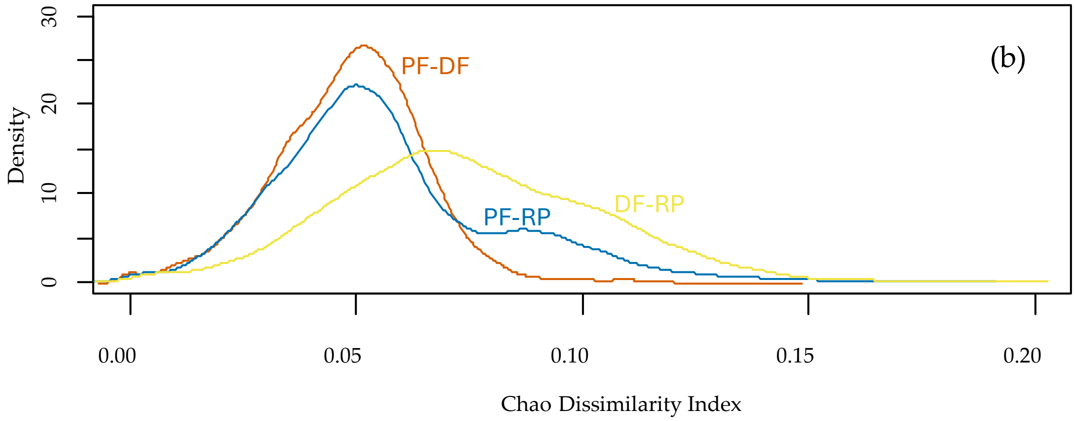

Supplement: Supplementary file 1 [file insects-16-00121-s001.zip › Revised_Supplementary Figure_S2.pdf]
